# Role of *Bifidobacterium breve* PRL2020 in reducing symptoms of antibiotic-associated diarrhea in children

**DOI:** 10.3389/fped.2026.1726821

**Published:** 2026-03-26

**Authors:** Alexander Bertuccioli, Annalisa Belli, Davide Sisti, Marco Bruno Luigi Rocchi, Chiara Maria Palazzi, Giordano Bruno Zonzini, Filippo Boccellari, Roberto Sacchetti, Francesco Di Pierro, Giuseppe Gregori

**Affiliations:** 1 Department of Biomolecular Sciences, University of Urbino Carlo Bo, Urbino, Italy; 2 Microbiota International Clinical Society, Torino, Italy; 3 Primary Care Pediatricians, AUSL Piacenza, Piacenza, Italy; 4 Scientific & Research Department, Velleja Research, Milano, Italy; 5 Department of Medicine and Technological Innovation, University of Insubria, Varese, Italy

**Keywords:** amoxicillin, antibiotic-associated diarrhea, *Bifidobacterium breve*, microbiota, probiotics

## Abstract

**Background:**

Antibiotic-associated diarrhea (AAD) is a common adverse effect of amoxicillin and amoxicillin/clavulanate therapy in children, primarily driven by antibiotic-induced dysbiosis, altered fermentation, and reduced production of short-chain fatty acids. Probiotics may help prevent AAD, but many strains are inhibited by these antibiotics. The present study specifically addresses the clinical impact of a single probiotic strain, *Bifidobacterium breve* PRL2020, rather than the intestinal microbiota as a whole.

**Methods:**

We conducted a 15-day, prospective, single-center controlled study enrolling children between January 2021 and December 2022, recruited by three Italian pediatricians from the Lombardy region (Northern Italy), to evaluate the efficacy of *Bifidobacterium breve* PRL2020, a strain with documented resistance to amoxicillin and amoxicillin/clavulanate, in children aged 4–12 years undergoing antibiotic therapy. Parents recorded daily bowel movements, episodes of diarrhea, abdominal pain (VAS), and stool consistency (Bristol scale).

**Results:**

Among 426 enrolled children (284 supplemented, 142 controls), supplementation with *B. breve* PRL2020 significantly reduced the number of diarrheal episodes (*p* < 0.001) and abdominal pain (*p* < 0.005) compared with controls, without affecting stool frequency or consistency. Some children experienced mild diarrheal events (<3 episodes/day) not meeting the formal definition of AAD but likely reflecting partial manifestations of AAD-related mechanisms.

**Conclusions:**

*B. breve* PRL2020 was associated with a significant reduction in antibiotic-associated diarrhea and abdominal discomfort in children receiving amoxicillin or amoxicillin/clavulanate. Its antibiotic resistance profile supports its use as a precision probiotic. Accordingly, the observed effects should be interpreted as strain-specific outcomes attributable to PRL2020 supplementation, and not as evidence of a global microbiota-wide modification. Longer-term, multi-omics studies are needed to confirm durability of the effect and to investigate combined probiotic–prebiotic strategies for optimizing gut microbiota resilience.

## 1 Introduction

Amoxicillin and amoxicillin/clavulanate represent widely used treatment options for a broad range of pediatric conditions—including acute otitis media, sinusitis, pharyngitis, and pneumonia—but are frequently associated with gastrointestinal adverse effects (1–4). In children treated with these antibiotics, adverse events such as vomiting, diarrhea, and rash occur with clinically relevant frequency, particularly in association with clavulanic acid (2–4). Among the gastrointestinal complications described in the literature, two main entities may occur: Drug-Induced Enterocolitis Syndrome (DIES) and Antibiotic-Associated Diarrhea (AAD) (5–7). DIES is a rare non–IgE-mediated hypersensitivity reaction affecting the gastrointestinal tract, characterized by delayed vomiting, abdominal pain, diarrhea, pallor, and, in severe cases, hypovolemic shock (6, 7). Although uncommon, DIES has been reported in association with amoxicillin or amoxicillin/clavulanate in both pediatric and adult case series (6–13). Its estimated incidence is approximately 0.4% among children evaluated for suspected hypersensitivity reactions (12). In contrast, nausea, vomiting, and diarrhea are more commonly recognized adverse effects of amoxicillin therapy (14).

AAD represents the most frequent gastrointestinal complication of oral penicillins (1). A pooled analysis of pediatric clinical trials reported an overall AAD incidence of 17.2%, reaching nearly 20% for amoxicillin/clavulanate and varying according to formulation ratios (1). The pathophysiology of AAD is multifactorial and involves antibiotic-induced disruption of the intestinal microbiota, impaired carbohydrate fermentation, osmotic imbalance, pathogen overgrowth, and reduced production of short-chain fatty acids (SCFAs) (1, 15–17). Decreased SCFA availability impairs colonic salt and water absorption, contributing to both osmotic and secretory diarrhea (17, 18). Additionally, the clavulanate component may increase intestinal motility, further promoting diarrheal symptoms (19). Antibiotics may also alter microbial diversity and metabolic profiles, including bifidobacterial populations, leading to persistent compositional changes even after treatment discontinuation (16, 20).

Given these mechanisms, probiotic supplementation has been proposed as a strategy to mitigate AAD risk (21–27). Several strains from the genera *Lactobacillus*, *Bifidobacterium*, *Saccharomyces*, *Bacillus*, *Clostridium*, and *Enterococcus* have demonstrated varying degrees of efficacy in reducing antibiotic-associated diarrhea in children (21–25). A Cochrane review and subsequent meta-analyses reported a moderate protective effect of probiotics, particularly at higher doses (≥5 billion CFU/day), although heterogeneity across strains and formulations remains substantial (22, 26, 27). Importantly, probiotic effects are strain-specific and cannot be generalized across species.

Most bifidobacterial strains are susceptible to amoxicillin–clavulanate; however, *Bifidobacterium breve* PRL2020 has been characterized for its *in vitro* resistance to amoxicillin and amoxicillin–clavulanate, as well as for genomic determinants underlying this phenotype (28, 29). In gut-simulating batch cultures, PRL2020 demonstrated persistence in the presence of amoxicillin–clavulanic acid and complex fecal microbiota (28). This strain-specific resistance profile aligns with the concept of precision bacterial therapy, already exemplified by the co-administration of rifaximin-resistant *Bifidobacterium longum* W11 with rifaximin (30).

Although no direct evidence currently supports probiotic prevention of DIES (5), bifidobacteria are known to contribute to mucosal and systemic immune tolerance (31), and early-life bifidobacterial colonization promoted, for example, by breastfeeding has been associated with beneficial immune imprinting (32).

Nevertheless, current clinical evidence regarding AAD prevention remains heterogeneous. For instance, a multispecies probiotic evaluated in a randomized clinical trial showed partial benefit in reducing overall diarrhea but did not significantly prevent strictly defined AAD (33).

Considering these considerations, the selection of an antibiotic-resistant, strain-specific probiotic represents a rational approach to mitigate AAD during *β*-lactam therapy.

The aim of this study was to evaluate the efficacy of *Bifidobacterium breve* PRL2020 in reducing the frequency of diarrheal episodes in children undergoing antibiotic treatment with amoxicillin or amoxicillin/clavulanic acid. The present investigation was designed to assess clinical endpoints related to the administration of this single probiotic strain. While microbiota-related mechanisms may represent a plausible explanatory framework, the study did not aim to provide a comprehensive assessment of microbiota-wide compositional changes, and any reference to microbial modulation should be interpreted within this strain-specific context.

## 2 Materials and methods

### 2.1 Study design

The study had a prospective and monocentric design with a control group, and participants were recruited between January 2021 and December 2022 by three Italian pediatricians from the Lombardy region (Northern Italy). The primary objective was to evaluate the efficacy of the supplement of *B. breve* (PRL2020) available in Italy under the brand name of Brevicillin® (Pharmextracta S.p.A.) in reducing the frequency of diarrheal episodes in children undergoing antibiotic treatment with amoxicillin or amoxicillin/clavulanic acid. For this purpose, the probiotic treatment was taken concurrently with the antibiotic therapy, as *Bifidobacterium breve* PRL2020 had been shown to be resistant to amoxicillin and amoxicillin–clavulanate. Exclusion criteria were applied by the individual pediatricians and consisted of the absence of chronic diseases, the presence of other diagnosed bacterial or viral pathologies in addition to the one requiring amoxicillin/clavulanic acid, and the use of other supplements containing microorganisms different from the one under investigation. Furthermore, cases in which the caregiver suffered from psychological or psychiatric disorders, or where linguistic and cultural barriers were present, were excluded. In the case of suspected psychiatric disorders, the pediatrician assessed, based on clinical experience, the possible presence of such conditions in the caregivers; in doubtful cases, exclusion was applied. Pediatric patients aged between 4 and 12 years were included; with the restriction to use only the proposed supplement as integration during the study period. The study lasted a total of 15 days. A daily questionnaire was filled in by the parents, based on the child's indications, in order to record: the total number of daily bowel movements, the number of episodes of diarrhea, and the severity of abdominal pain assessed using a VAS scale. Stool consistency was objectively assessed by the parent using the Bristol Stool Scale provided at the beginning of the study.

### 2.2 Product characteristics

Each child in the treatment group received the supplement Brevicillin (Pharmextracta S.p.A., authorization no. 159330), containing 2 × 10^10^ CFU/stick of *Bifidobacterium breve* PRL2020. Each stick was dissolved directly in the mouth, without the addition of liquids. Pediatricians recommended administering the probiotic powder in the evening, as the last activity before going to bed; Brevicillin should in fact be taken on an empty stomach (away from meals). Consequently, it was advised to take it approximately two hours after the last meal, preferably in the evening. It is important to note that the pediatricians reminded the parents about the product storage in the refrigerator. Refrigeration preserved the quality of the product and ensured the viability of the probiotics. For this reason, it was recommended to avoid exposure to heat sources or temperature fluctuations, to store the product protected from light and preferably at a temperature between +2 °C and +8 °C, and to transport it at temperatures below 25 °C for periods not exceeding 48 h.

### 2.3 Statistical analysis

**Figure 1:**
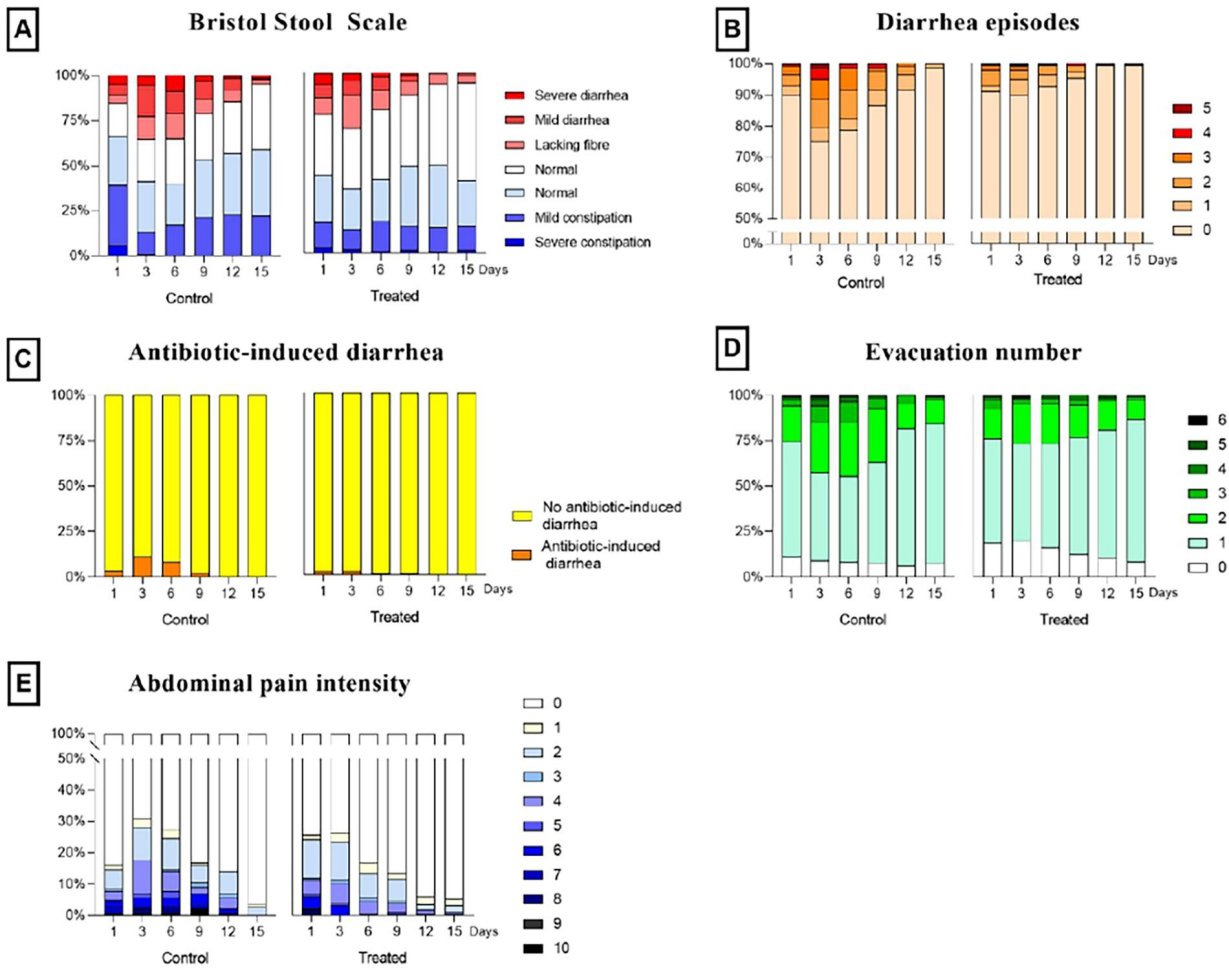
Trends of Bristol Stool Scale **(A)**, diarrhea episodes **(B)**, antibiotic-induced diarrhea **(C)**, evacuation number **(D)** and abdominal pain intensity **(E)** are shown. Days 1,3,6,9,12,15 are chosen as references. In the left part of each box, trends for control groups are shown, while in the right part treated group categories are reported.

To evaluate the temporal trends of the considered variables according to time and treatment group (treated vs. control), a time-dependent modeling approach was applied. Two GLM models for repeated measures were applied, specifically a multivariate MANOVA. Two models were required since the Bristol Stool Scale values were, of course, recorded only in the presence of at least one episode of diarrhea per day, making it necessary to analyze this parameter separately with repeated measures ANOVA. An additional variable was also created, representing the presence or absence of antibiotic-induced diarrhea, classified according to the guidelines as more than three episodes of loose or watery stools within 24 h (1) (34). In addition to this predefined criterion, exploratory analyses were conducted considering a threshold of ≥2 episodes/day to assess potential early or subclinical gastrointestinal alterations and to evaluate the discriminative capacity between treated and control groups. This secondary threshold was analyzed for interpretative purposes and did not replace the formal AAD definition. The section “other pathologies” includes conditions such as bronchitis, pharyngitis, pharyngotonsillitis, and sinusitis. The dependent variables of the first model included repeated daily measurements over 15 days: presence/absence of antibiotic-induced diarrhea, number of daily defecations, number of daily diarrheal discharges and abdominal pain assessed using a VAS scale (1–10). The VAS consisted of a 100-mm horizontal line anchored at each end with verbal descriptors representing the extremes of the sensation being measured. For abdominal pain assessment, these anchors were labeled as “no pain” on the left and “worst pain imaginable” on the right. Children were instructed to mark a point on the line that corresponds to their current level of pain. The dependent variable considered in the second model, the repeated measures ANOVA model, was the Bristol Stool Scale score. Age in years and number of days of antibiotic treatment were included as covariates in both models. Time was treated as a within-subject factor, while group was included as a between-subject factor. Interactions between time and group, time and antibiotic days, and time and age were also included in the models. This modeling framework allowed us to account for within-subject correlations over time. Moreover, it enabled the identification of potential interactions between temporal trends and clinical or demographic variables. Moreover, *post-hoc* contrast analyses were performed using the simple contrast method, with day 1 set as the reference, in order to highlight significant differences between the reference day and each subsequent day of the study, day by day. Results were presented graphically using heatmaps, in which only the boxes with significant *p-values* are highlighted (Figure 1). In addition, t-tests were used to investigate differences in quantitative variables between the treated and control groups (age, number of doses per day, duration of antibiotic treatment, and number of daily evacuations at baseline). The significance level was set at alpha = 0.05, and all statistical analyses were conducted using R studio (version 4.4.1) and Excel.

## 3 Results

### 3.1 Participants

The study included a total of 426 children aged 4 to 12 years, recruited between January 2021 and December 2022 by three Italian pediatricians from the Lombardy region (Northern Italy), allocated to the treatment group (*n* = 284; 66.7%) and the control group (*n* = 142; 33.3%). The treatment group comprised 147 females (51.8%), while the control group included 65 females (45.8%). The mean age was 6.7 ± 2.7 years in the treatment group and 6.5 ± 2.7 years in the control group. Regarding antibiotic therapy, 131 children (30.7%) received amoxicillin, and 295 children received amoxicillin + clavulanic acid (69.3%) for an average duration of 8.0 ± 1.6 days (MIN-MAX: 5–10 days), with 2 to 3 doses per day. To assess bowel movements at T0, parents were asked to report the child's daily evacuation frequency, which resulted in an average of 1.1 ± 0.4 evacuations per day (MIN-MAX: 0–3 evacuations).

Groups were comparable and showed similar characteristics, since no significant differences were found in terms of age in years (t = −0.623; *p* = 0.553), number of antibiotic doses (t = 0.561; *p* = 0.575), duration of antibiotic treatment (t = −1.377; *p* = 0.169) and daily evacuations at baseline, as highlighted in Table 1.

**Table 1:** Descriptive statistics of baseline condition. All values are expressed as mea*n* ± standard deviation.

Baseline variable	Control	Treated	t (p)
Age (years)	6.51 ± 2.67	6.68 ± 2.70	−0.623 (0.553)
Nr. Doses/die	2.41 ± 0.49	2.38 ± 0.49	0.561 (0.575)
Duration of antibiotic treatment (days)	7.85 ± 1.62	8.04 ± 1.54	−1.377 (0.169)
Daily evacuations at baseline	1.08 ± 0.30	1.12 ± 0.42	−0.889 (0.374)

Considering the overall observation period, the proportion of diarrhea episodes was 6.34% in the treated group vs. 20.85% in the control group, indicating a lower incidence of diarrhea episodes in the treated group.

In Figure 1, the trends of the measured variables were shown at different reference time points (days 1,3,6,9,12,15). According to the Bristol Stool Scale (Figure 1A), subjects in the treated group progressively shifted toward normal stool consistency, while the control group exhibited more variability and a higher proportion of diarrheic or constipated stools. The frequency of diarrhea episodes markedly decreased in the treated group, with most subjects reporting no episodes after day 6, whereas occasional cases persisted in the control group (Figure 1B). Similarly, antibiotic-induced diarrhea was almost absent among treated subjects, suggesting a protective effect of the treatment (Figure 1C). Regarding the number of evacuations, the treated group maintained a more stable and physiological frequency (2–3 evacuations per day), with fewer occurrences of both low and high evacuation rates compared with controls (Figure 1D). Finally, abdominal pain intensity declined over time in both groups, but the reduction was more pronounced in treated subjects, among whom the percentage of participants reporting no pain progressively increased (Figure 1E). Overall, the treatment appeared to promote intestinal regularity, stool normalization, and reduced gastrointestinal discomfort, indicating a beneficial effect on gut health during the 15-day period.

### 3.2 General linear model

The within-subjects effects analysis revealed significant values for the time*group interaction (F = 2.407, *p* < 0.001), indicating that the trend of the dependent variables differed over time with a significant influence of group membership. The time*antibiotic days interaction term was nearest to the significance level (F = 1.300, *p* = 0.09), suggesting a possible effect of time on the duration of antibiotic treatment. No significant effects were observed for the time*age (F *=* *1.05, p* *=* *0.382)* interaction, suggesting that the effect of time on the evaluated symptoms was not dependent on age. Temporal patterns of subjects with respect to age appeared stable, indicating that children showed the same type of change over time regardless of sex or age. The main effect of time alone was not significant (F = 1.015, *p* = 0.451), indicating the absence of a common pattern of change across all children. The lack of a main time effect suggests that, on average, the measures did not significantly change over time when considering all subjects together. However, the significant time*group interaction demonstrated that trends over time varied according to group membership, pointing to a treatment-related effect. When focusing on the effect of group membership on individual symptoms, all evaluated symptoms were significantly dependent on group: number of diarrheal discharges (F = 13.147, *p* < 0.001), number of daily defecations (F = 7.761, *p* < 0.001), antibiotic-induced diarrhea (F = 10.969, *p* < 0.001) and abdominal pain (F = 7.232, *p* < 0.001). None of these symptoms were significant with respect to the main effect of time, again suggesting a clear effect of the proposed treatment.

Post-hoc analyses revealed significant differences between groups for specific symptoms. In particular, children in the treatment group reported statistically lower mean values of diarrhea episodes (*p* < 0.001, *Δ*_T−C_ = −0.231, 95% CI: −0.286; −0.177), antibiotic-induced diarrhea (*p* < 0.001, D_T−C_ = −0.037, 95%CI: −0.046; −0.027), number of daily evacuations (*p* < 0.001, *Δ*_T−C_ = −0.230, 95%CI: −0.326; −0.135) and abdominal pain (*p* < 0.005, *Δ*_T−C_ = −0.329, 95% CI: −0.502; −0.155) compared to the control group. By contrast, no relevant differences were observed between groups for the stool consistency assessed by the Bristol Stool Scale (*p* = 0.653, *Δ*_T−C_ = −0.058, 95% CI: −0.313; 0.197). These results suggest that the treatment effect was mainly evident in relation to both diarrhea and antibiotic-induced diarrhea, abdominal pain and number of daily evacuations, whereas it did not appear to significantly affect stool consistency.

**Figure 2:**
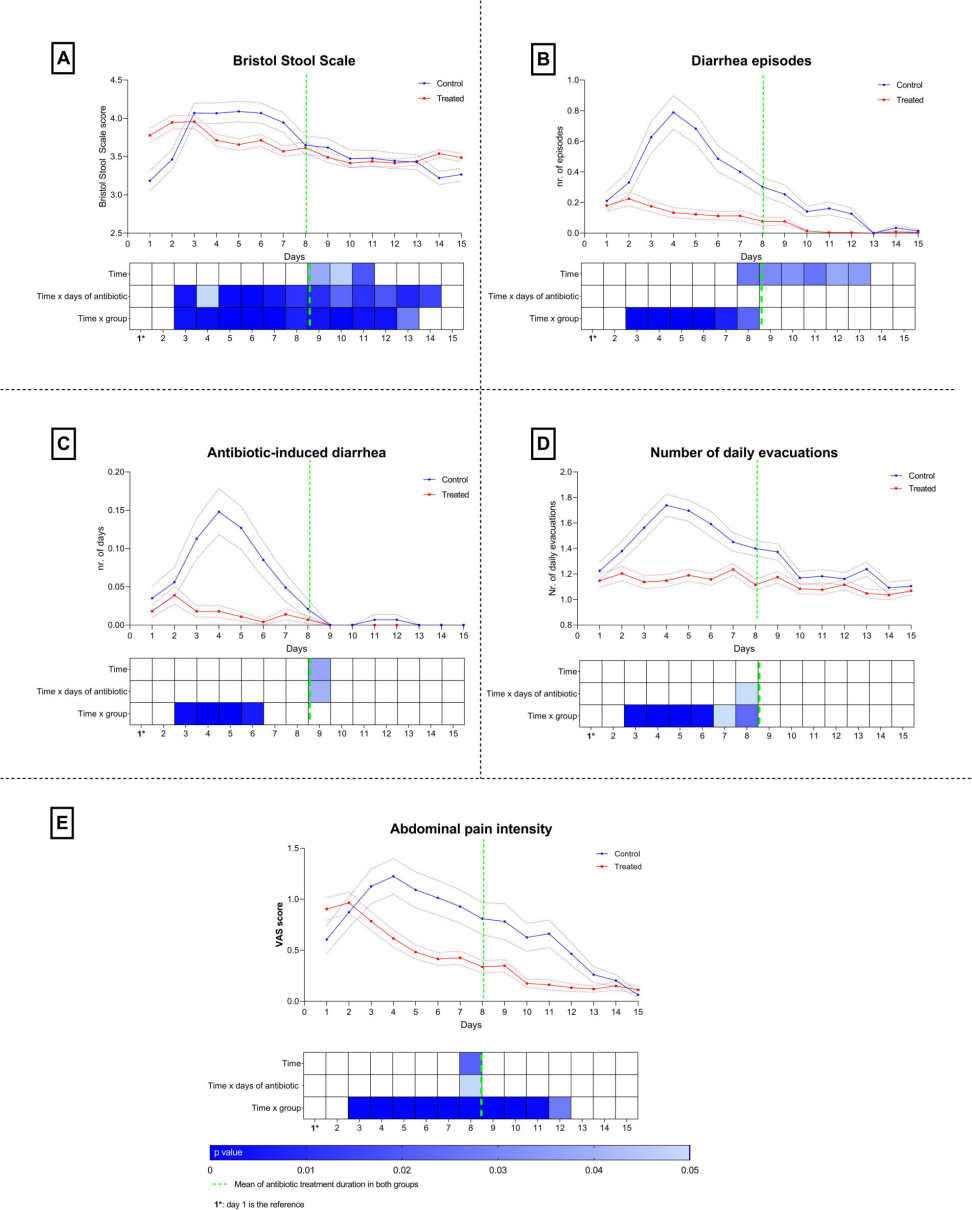
Temporal trends and average course of the perceived symptoms are displayed in the upper part of each panel. Solid lines represent the mean (red line for the treated group, blue line for the control group), while dashed lines indicate the 95% confidence intervals (lower and upper limits). In the lower part of each panel, heatmaps illustrate the significance levels of the within-subjects contrast tests for the time effect and the interaction terms time × days of antibiotic treatment and time × group. Only boxes with significant values are colored, with more intense colors indicating lower p-values. All contrasts were performed using day 1 as the reference. Trends of Bristol Stool Scale **(A)**, diarrhea episodes **(B)**, antibiotic-induced diarrhea **(C)**, number of daily evacuations **(D)**, Abdominal pain intensity **(E)** are shown.

 Figure 2 shows the temporal trend of the perceived symptoms during the study period (upper part of each panel). In both groups, a slight increase in symptom intensity was observed during the first two days. From day 2 onwards, the trajectories of the treated and control groups separate, with the treated group presenting consistently lower values. Around day 8, the two trends appear to converge again, precisely at the end of the antibiotic treatment for both groups. This leads us to consider *Bifidobacterium breve* PRL2020 as a potential “temporary shield” during the phase of antibiotic-induced dysbiosis, capable of helping to reduce the symptoms associated with antibiotic therapy itself. Upon discontinuation of the antibiotic, the microbiota tends to recover physiologically, and the probiotic's effectiveness appears less evident during this phase.

## 4 Contrast comparisons

In order to specifically compare the trend of individual variables over time, a within-subjects contrast test was performed using the simple contrast metric, with reference to group 1. For the interaction term time  ×  age, no significant contrasts were found, highlighting the absence of meaningful changes in the measured parameters between baseline and the study period, suggesting effects independent of the children's age. By analyzing the simple contrasts using group 1 as the reference, some peculiarities were highlighted. The interaction contrast time  ×  days of antibiotic treatment was significant for the comparison between day 1 and days 8–9, indicating that: the effect of antibiotic treatment duration on diarrhea (p_day8_ < 0.05) (Figure 2B), antibiotic-associated diarrhea (p_day9_ < 0.05) (Figure 2C), daily number of evacuations (p_day8_ < 0.05) (Figure 2D), and abdominal pain (p_day8_ < 0.05) (Figure 2E) appeared to manifest in a differentiated way only around the eighth day compared to baseline, except for changes in the Bristol Stool Scale (Figure 2A), where differences from baseline are already evident from day 3 (p_day3_ < 0.05) up to day 14 (p_day14_ < 0.05). These timings correspond exactly to the average duration of antibiotic treatment in both groups, suggesting that stool consistency, as measured by the Bristol Stool Scale, is significantly different from baseline for almost the entire observation period, whereas the other symptoms started to improve only at the end of the antibiotic treatment (day 8). An interesting aspect emerges from the analysis of the contrasts for the time effect: also in this case, changes started to appear around day 8, specifically from day 8 to day 13 for the presence of diarrhea (p_day8_ < 0.05; p_day13_ < 0.05) (Figure 2B), from day 9 to day 11 for stool consistency (p_day9_ < 0.05; p_day11_ < 0.05) (Figure 2A), on day 9 for antibiotic-associated diarrhea (p_day9_ < 0.05) (Figure 2C), and on day 8 for abdominal pain (p_day8_ < 0.05) (Figure 2E). Analyzing the time  ×  group effect, significant periods of improvement compared to baseline were observed, specifically: from day 3 to day 13 for both stool consistency (p_day3_ < 0.01; p_day13_ < 0.05) (Figure 2A) and perceived abdominal pain (p_day3_ < 0.01; p_day13_ < 0.05) (Figure 2E); from day 3 to day 8 for diarrhea (p_day3_ < 0.001; p_day8_ < 0.05) (Figure 2B); from day 3 to day 6 for both antibiotic-associated diarrhea (p_day3_ < 0.01; p_day6_ < 0.05) (Figure 2C) and the number of daily evacuations (p_day3_ < 0.01; p_day8_ < 0.05) (Figure 2D).

The temporal trends displayed in the upper panels of Figure 2, indicated that all gastrointestinal symptoms increased during the initial days of antibiotic treatment, with consistently higher values in the control group compared to the treated group. The Bristol Stool Scale, number of daily evacuations, diarrhea episodes, antibiotic-induced diarrhea, and abdominal pain intensity all peaked between days 3 and 5, with a gradual decrease thereafter. Around day 8, the two trends appeared to converge again, precisely at the end of antibiotic treatment in both groups. The heatmaps in the lower panels illustrated the significance levels of the within-subject contrast tests. Significant time effects were observed particularly between days 3 and 8, when symptom variations were most pronounced. The *time  ×  group* interaction was significant mainly during the central phase of treatment, highlighting a greater and earlier reduction of symptoms in the treated group compared with controls.

This suggests that, over time and at the end of antibiotic administration, symptoms tend to improve spontaneously compared to the initial treatment period. However, as highlighted by the analysis of the time  ×  group effect, the probiotic appears to anticipate symptom improvement, which begins as early as day 3, as shown in Figure 2. The absence of significant contrasts between day 1 and day 2 in all considered effects, may suggest a colonization period (24–48 h) required for the supplement to begin exerting its beneficial effects. The term “colonization” is used here to indicate an onset-of-action timeframe; dedicated strain-tracking studies would be required to demonstrate *in vivo* colonization or long-term persistence.

## 5 Discussion

Although AAD is typically defined as three or more episodes of loose or watery stools within 24 h or over two or more consecutive days (1) (34), not all gastrointestinal events meet this criterion; nonetheless, considering the mechanisms described in the literature, such events may reflect alterations of the gut microbiota or transient functional impairments of intestinal dynamics. While the formal definition of AAD requires ≥3 episodes per day, our data suggest that a threshold of ≥2 episodes/day already displays a relevant discriminative capacity between treated and control groups. This finding may indicate that even subthreshold diarrheal manifestations reflect early stages of antibiotic-induced dysbiosis and are clinically meaningful.

The findings of this study confirm that supplementation with *Bifidobacterium breve* PRL2020 in children receiving antibiotic therapy with amoxicillin or amoxicillin/clavulanate is associated with a significant reduction in episodes of antibiotic-associated diarrhea (AAD) and abdominal pain, without significantly affecting stool frequency or consistency. These results are consistent with the literature showing that AAD is one of the most frequent complications of pediatric antibiotic therapy (1, 15, 16, 19) and that probiotics represent an effective strategy for its prevention (26, 27). From a microbiological standpoint, the plausibility of PRL2020 as a co-administered strain is supported by previous *in vitro* evidence showing its ability to persist in gut-simulating batch cultures in the presence of amoxicillin–clavulanate and a complex fecal microbiota. In addition, strain-specific characterization studies have provided genomic context to the antibiotic-resistance phenotype of PRL2020, supporting its selection for clinical use during *β*-lactam exposure.

The distinctive feature of *B. breve* PRL2020 lies in its documented resistance to amoxicillin and amoxicillin–clavulanate (28, 29), which allows concomitant administration with these antibiotics while maintaining the viability of the strain; consistently, in gut-simulating *in vitro* batch cultures with a complex fecal microbiota, *B. breve* PRL2020 was shown to persist even in the presence of amoxicillin–clavulanic acid (28). This evidence supports the concept of strain-specific ecological fitness and persistence under antibiotic pressure, which may be particularly relevant for co-administration approaches during amoxicillin–clavulanate therapy. This property aligns with the concept of precision bacterial therapy, already demonstrated in other clinical settings using probiotic strains resistant to specific antibiotics (30). Such an approach may represent a model for managing iatrogenic dysbiosis, preventing the loss of probiotic efficacy during antimicrobial treatment.

Our study further supports the hypothesis that modulation of the intestinal microbiota may have beneficial effects not only on the prevention of AAD but also on the overall resilience of the intestinal ecosystem following antibiotic exposure. Thus, any reference to “microbiota modulation” should be interpreted as a mechanistic hypothesis consistent with prior strain-level evidence, rather than as a direct claim of broad microbiota restructuring.

Antibiotics are known to profoundly disrupt microbial diversity and key metabolites such as short-chain fatty acids (SCFAs), thereby promoting the overgrowth of opportunistic pathogens (15, 16). In this scenario, specific probiotics may help to re-establish a more stable and functional microbial community. Future multi-omics approaches will be needed to determine whether PRL2020 supplementation is associated with measurable compositional and functional shifts at the ecosystem level. Our findings also suggest that *Bifidobacterium breve* PRL2020 may offer the greatest benefit during the acute phase of antibiotic-induced dysbiosis, acting as a temporary “shield.” This effect is intended as a clinical metaphor for the early symptom reduction observed during antibiotic exposure, likely linked to PRL2020 strain-specific persistence under antimicrobial pressure. Once antibiotic therapy is discontinued, the probiotic's effect may nevertheless appear less evident, reflecting the physiological recovery of the intestinal microbial community. Notably, SCFA-related considerations refer to general AAD pathophysiology, as strain-specific SCFA production by PRL2020 was not investigated in the present clinical study. Given the above, the clinical rationale for testing PRL2020 is primarily related to its strain-level features (including antibiotic resistance and persistence in simulated intestinal ecosystems), rather than to generic assumptions applicable to the species *B. breve* or to probiotics as a whole.

It is important to emphasize that episodes of diarrhea recorded in this study do not necessarily correspond to overt cases of AAD. The classical definition of AAD requires ≥3 loose or watery stools per 24 h (1); in our cohort, some children experienced milder symptoms (e.g., 1–2 episodes/day) that did not meet this formal criterion but may still represent partial manifestations of AAD pathophysiology. As described in the literature, the mechanisms underlying AAD include clavulanate-induced stimulation of peristalsis, impaired carbohydrate fermentation with osmotic accumulation, reduced SCFA production, and dysbiosis favoring the proliferation of opportunistic pathogens (1, 15, 16). Subclinical involvement of one or more of these components could plausibly lead to mild diarrheal episodes not meeting the formal diagnostic threshold for AAD, yet still clinically relevant.

Beyond probiotics, several prebiotic strategies have been proposed to maintain the intestinal microbiota, including fermentable fibers, polyphenols, and other selective substrates (35, 36). In particular, Cazzaniga et al. (35) highlighted the potential role of nutraceuticals such as berberine in modulating the microbiota and metabolic disorders, while Bertuccioli et al. (36) provided evidence that targeted prebiotic approaches can help sustain microbial composition even under pathological conditions. These observations suggest that an integrated intervention—combining selected probiotics with specific prebiotic substrates—could enhance microbial resilience and reduce the risk of post-antibiotic gastrointestinal complications. These approaches are reported as complementary perspectives from the literature and were not evaluated within the present study design.

Another area of interest is the possible protective effect against non–IgE-mediated reactions, such as Drug-Induced Enterocolitis Syndrome (DIES) (6, 7, 13). Although there is currently no definitive clinical evidence that probiotics can prevent DIES, the ability of bifidobacteria to promote mucosal and systemic immune tolerance (31) deserves further investigation.

## 6 Study limitations

This work has some limitations that must be considered when interpreting the findings. First, the single-center design and the relatively short duration (15 days) do not allow assessment of long-term effects on microbiota stability or the possible recurrence of AAD. Moreover, no metagenomic analyses were performed to describe in detail the microbial compositional changes, nor was there functional characterization of microbial metabolites (e.g., SCFAs). In addition, strain-tracking analyses were not performed to directly assess PRL2020 engraftment or persistence *in vivo*. Finally, despite the adequate sample size, recruitment from a single geographic area limits the generalizability of the results to populations with different diets or genetic backgrounds. A possible additional limitation of the study is the heterogeneity of the recruitment period and the inter-pediatric variability in case selection.

## 7 Future perspectives

To consolidate and extend these findings, future studies should:

1. include longer follow-up periods to assess the persistence of the protective effect and the long-term impact on the microbiota;
2. integrate multi-omics approaches (metagenomics, metabolomics) to correlate clinical outcomes with precise microbial and metabolic changes;
3. compare *B. breve* PRL2020 with other antibiotic-resistant probiotic strains or synbiotic formulations, also assessing the addition of specific prebiotics suggested by recent literature (35, 36);
4. broaden recruitment to different age groups and geographic settings to validate reproducibility across diverse populations.

In conclusion, our results confirm the efficacy of *B. breve* PRL2020 in significantly reducing the incidence of antibiotic-associated diarrhea in children and highlight the potential of a precision-medicine approach that combines antibiotic-resistant probiotics and, prospectively, targeted prebiotic interventions to preserve intestinal microbiota balance during and after antibiotic therapy. In this context, “microbiota balance” refers to a clinically oriented concept based on symptom prevention; microbiota-wide confirmation will require dedicated metagenomic/metabolomic evaluation. Future investigations integrating strain tracking, metagenomic profiling, and metabolomic endpoints will be essential to clarify whether PRL2020 supplementation translates into measurable microbiota-wide changes and specific functional outputs *in vivo*.

## 8 Conclusions

Supplementation with *Bifidobacterium breve* PRL2020 during antibiotic treatment with amoxicillin or amoxicillin/clavulanate significantly reduced the incidence of diarrhea and abdominal pain in children without affecting stool frequency or consistency. The strain's intrinsic resistance to these antibiotics supports its use in combination therapy and represents a step toward precision probiotic strategies. Broader, long-term, and multi-omics studies are warranted to confirm these findings, clarify the underlying mechanisms, and explore combined probiotic–prebiotic approaches for enhancing intestinal microbiota resilience during antibiotic exposure. These findings should be interpreted as PRL2020 strain-specific and should not be generalized to other *B. breve* strains or bifidobacterial species, as probiotic functional properties are inherently strain-dependent.

## Data Availability

The original contributions presented in the study are included in the article/Supplementary Material, further inquiries can be directed to the corresponding author.
